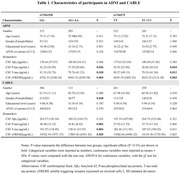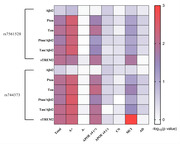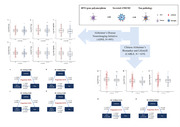# sTREM2 mediates the correlation between BIN1 gene polymorphism and tau pathology in Alzheimer’s disease

**DOI:** 10.1002/alz.087956

**Published:** 2025-01-03

**Authors:** Fan Guo, Ze‐Hu Sheng, Ya‐nan Ou, Zuo‐teng Wang, Qing‐Fei Zhao, Tan Meng Shan, Lan Tan, tanlan

**Affiliations:** ^1^ Department of Neurology, Qingdao Municipal Hospital, Qingdao university, Qingdao, Shandong China; ^2^ Department of Neurology, Qingdao Municipal Hospital, Qingdao University, Qingdao, Shandong China; ^3^ Department of Geriatrics, The First Affiliated Hospital of Chongqing Medical University, Chongqing Medical University, Chongqing, Chongqing China; ^4^ Qingdao Municipal Hospital, Qingdao University, Qingdao China; ^5^ Qingdao Municipal Hospital, Qingdao University, qingdao, Shandong China; ^6^ Qingdao Municipal Hospital, Qingdao university, Qingdao, Shandong China; ^7^ Qingdao Municipal hospital, Qingdao university, Qingdao, Shandong China

## Abstract

**Background:**

Bridging integrator 1 (*BIN1*), one of the most strongly associated gene with Alzheimer’s disease (AD). It has been reported to play a role in the pathological processes of AD; however, the exact mechanism has not yet been completely found.

**Method:**

Alzheimer’s Disease Neuroimaging Initiative (ADNI, N = 495) was the discovery cohort, and the Chinese Alzheimer’s Biomarker and LifestylE (CABLE, N = 619) study was used to replicate the results. Two *BIN1* gene polymorphism (rs7561528 and rs744373) were included in the analysis. Multiple linear regression model was used to examine the *BIN1* loci relationship with cerebrospinal fluid (CSF) AD biomarkers and alternative biomarker of microglial activation microglia‐soluble triggering receptor expressed on myeloid cells 2 (sTREM2). Causal mediation analysis was conducted through 10,000 bootstrapped iterations to explore the mediating effect of sTREM2 on AD pathology.

**Result:**

In the ADNI database, we found a significant association between *BIN1* loci (rs7561528 and rs744373) and the levels of CSF phosphorylated‐tau (P‐tau) (*Pc* = 0.017; 0.010, respectively) and total‐tau (T‐tau) (*Pc* = 0.011; 0.013, respectively). The *BIN1* loci were also found to be correlated with the levels of CSF sTREM2 (*Pc* = 0.010; 0.008, respectively). Mediation analysis demonstrated that CSF sTREM2 partially mediated the association of the *BIN1* loci with P‐tau (rs7561528: Prop = 20.9% of total effect; rs744373: Prop = 24.9% of total effect) and T‐tau (rs7561528: Prop = 36.6% of total effect; rs744373: Prop = 44.1% of total effect). The analysis in CABLE study replicated the mediation role of rs7561528.

**Conclusion:**

This study demonstrated correlation between *BIN1* loci and CSF AD biomarkers as well as microglia biomarker, particularly in preclinical AD. Additionally, the link between *BIN1* loci and AD pathology was partially mediated by CSF sTREM2